# Modeling the Decision of Mental Health Providers to Implement Evidence-Based Children’s Mental Health Services: A Discrete Choice Conjoint Experiment

**DOI:** 10.1007/s10488-017-0824-z

**Published:** 2017-09-16

**Authors:** Charles E. Cunningham, Melanie Barwick, Heather Rimas, Stephanie Mielko, Raluca Barac

**Affiliations:** 1 Patient Centered Health Care, Department of Psychiatry and Behavioural Neurosciences, Faculty of Health Sciences, Michael G. DeGroote School of Medicine, McMaster University, Hamilton Health Sciences, Hamilton, ON Canada; 2 CHES Research Institute, Peter Gilgan Centre for Research and Learning, The Hospital for Sick Children, Toronto, Canada; 3 Child and Youth Mental Health Research Unit, The Hospital for Sick Children, Toronto, Canada

**Keywords:** Preferences, Evidence-based practices, Implementation, Discrete choice experiment

## Abstract

Using an online, cross sectional discrete choice experiment, we modeled the influence of 14 implementation attributes on the intention of 563 providers to adopt hypothetical evidence-based children’s mental health practices (EBPs). Latent class analysis identified two segments. Segment 1 (12%) would complete 100% of initial training online, devote more time to training, make greater changes to their practices, and introduce only minor modifications to EBPs. Segment 2 (88%) preferred fewer changes, more modifications, less training, but more follow-up. Simulations suggest that enhanced supervisor support would increase the percentage of participants choosing the intensive training required to implement EBPs. The dissemination of EBPs needs to consider the views of segments of service providers with differing preferences regarding EBPs and implementation process design.

## Introduction

Systematic reviews support the effectiveness of an increasing number of treatments for child and youth mental health problems (Chorpita et al. 2011). Front line organizations and service providers, however, often fail to adopt, fully implement, or sustain potentially effective programs (Beidas and Kendall 2010; Stirman et al. 2012). Adherence varies considerably with administrators, supervisors, or service providers tailoring content to local contexts, altering the delivery process, adding or removing components of the intervention, rearranging the sequencing of sessions, adjusting the duration or pace of a program, or attempting to integrate the elements of different approaches (Palinkas et al. 2013; Stirman et al. 2013).

A growing body of evidence supports the conclusion that the implementation process is critical to the short-term outcome, long-term maintenance and ultimate value of evidence-based children’s mental health practices (EBP)s (Beidas and Kendall 2010). The decision to adopt and implement EBPs reflects a complex set of contextual, organizational, and individual factors (Aarons et al. 2011; Damschroder et al. 2009; Schoenwald and Hoagwood 2001; Wisdom et al. 2014). At the organizational level, limitations in the funding available for training, supervision, and long-term support, competing organizational demands, and turnover among staff and supervisors constitute barriers to the implementation of EBPs (Beidas et al. 2016). Service providers in organizations perceived to have more constructive cultures, to be more open to innovation, and less stressful report more positive attitudes toward the adoption of EBPs (Aarons and Sawitzky 2006).

Mental health practitioners are an integral part of the inner social context influencing the decision to adopt and implement evidence-based children’s mental health services (Aarons et al. 2012; Damschroder et al. 2009; Wisdom et al. 2014). Practitioner allegiance to competing therapeutic modalities, confidence in existing approaches to practice, attitudes regarding manualized models, access to expert supervision, concern regarding increased reporting requirements, difficulty integrating EBPs into work processes, or familiarity with the technology required may influence the adoption and implementation of EBPs (Beidas et al. 2016; Reid et al. 2013).

Given the pivotal role that practitioners play in the introduction of EBPs, their views regarding the design of the implementation process are likely to influence their engagement, participation, support for the implementation efforts of colleagues, and long-term commitment to newly introduced EBPs. Aarons et al. (2004) developed a 15-item measure of service provider attitudes regarding the adoption of EBPs. Attitudes regarding the adoption of EBPs were influenced by the initial appeal of a new approach, openness to innovation, the extent to which service providers were required to adopt new practices, and divergence between an EBP and existing approaches (Aarons 2004; Aarons et al. 2010). A 35-item addition to this measure (Aarons et al. 2012) suggested that the attitudes of children’s mental health service providers were also influenced by the perceived limitations of EBPs (e.g., simplicity or lack of applicability to complex problems), the extent to which EBPs were judged to fit the needs of clients, were consistent with the therapeutic orientation of service providers, provided the opportunity to practice without monitoring, and to balance the art and science of intervention. Organizationally, attitudes were influenced by the time and administrative demands of EBPs, organizational support, supervisory feedback, and the extent to which EBPs contributed to job security.

Cross sectional studies find an association between attitudes regarding EBP and self-reported utilization. Therapists with more positive attitudes regarding new therapeutic approaches, for example, are more likely to report the use of cognitive-behavioral strategies (Beidas et al. 2015). In a sample of 214 service providers, formal training in EBPs, positive attitudes toward EBP research, and perceptions regarding organizational openness to EBPs predicted greater self-reported utilization (Nelson and Steele 2007). Similarly, a study of 347 therapists found that, controlling for views regarding EBPs in general, the appeal of specific EBPs was associated with self-reported use of those approaches (Reding et al. 2014).

Palinkas and colleagues used ethnographic methods to study the short-term application and long-term intent to use EBPs introduced in the context of the Child STEPS project, an RCT of EBPs for children and youth with depression, anxiety, or conduct problems (Palinkas et al. 2008). Although some clinicians intended to discontinue the use of the EPBs once the trial was completed, most planned to employ components of the interventions included in the trial. A short period of time between training and application, an enthusiastic commitment to participation, and assignment to a modular condition allowing a more flexible application of the components of EBPs encouraged the long-term intent to use EBPs (Palinkas et al. 2008).

Subsequent studies suggest that training, supervisory support, and a successful experience implementing EPBs can contribute to a favorable shift in attitudes and sustained application of the components of EBPs (Chorpita et al. 2015; Palinkas et al. 2013). In three-month post trial interviews with therapists who participated in the Child STEPS study, for example, most (68%) applied components of EBP to work with non-study clients. Of these, 92% adapted EBPs in an effort to achieve an intervention that was more acceptable to youth or parents, enhanced alignment with organizational policies and clinic demands, or was more consistent with a therapist’s philosophical approach to clinical work (Palinkas et al. 2013). Quantitative follow-ups showed that therapists in the trial’s modular condition valued the responsiveness of an approach that provided therapists with flexibility in selecting the elements of EBPs (Chorpita et al. 2015).

## Methodological Gaps in the Study of EBP Implementation Preferences

There are several methodological gaps in studies examining attitudes regarding the implementation of EBPs. First, although the conceptual frameworks emerging from implementation studies have made an important contribution to research in this area (Aarons et al. 2011; Damschroder et al. 2009; Schoenwald and Hoagwood 2001; Wisdom et al. 2014), “… they provide a necessary but not sufficient guide for selecting and tailoring implementation strategies” (Powell et al. 2015). Determinant models (Nilsen 2015), reflect the individual factors that might influence the implementation of EBPs (Aarons et al. 2011; Damschroder et al. 2009; Schoenwald and Hoagwood 2001). In publicly funded children’s mental health services with competing demands on finite resources, however, implementation choices confront decision makers with tradeoffs regarding the relative importance of these individual factors. For example, although extended training and supervision may enhance skill acquisition, increase adherence, and support long-term implementation (Beidas and Kendall 2010), administrators may be concerned that a more intensive approach to implementation will reduce the time devoted to routine clinical care (Aarons et al. 2011). There is a need for methods that study implementation decisions in the context of the tradeoffs that influence real-world planning (Powell et al. 2015).

Second, when confronted with complex choices regarding EBPs, the decision strategies professionals apply are likely to vary greatly (Hauser 2014). Individuals might weigh the incremental contribution of a large number of EBP attributes or reduce decision complexity by adopting a set of simplifying heuristics (Hauser 2014). Simplifying strategies might include narrowing the hundreds of EBPs available to a consideration set that would be examined carefully or setting thresholds at which an option would be rejected (e.g. training time demands) (Hauser 2014). Decision making heuristics may vary as a function of choice complexity (Swait and Adamowicz 2001) or the investment of individual decision makers in the choices at hand (Peschel et al. 2016). The important role which decision making heuristics may play in choices regarding the implementation of EBPs suggests a need to develop approaches to the study of design preferences which activate the heuristics influencing real-world decisions.

Third, change models emphasize individual differences in readiness to implement new technologies (Rogers 2003). The diffusion of innovation theory proposed by Rogers, for example, recognizes variation in the rate at which individuals adopt innovative practices. Adoption is described by an S shaped logistic function predicting that diffusion begins slowly as innovators and early adopters embrace new technologies, increases exponentially as early and late majority adopters come on board, and slows or stops with a small group who are last to adopt a given innovation (Rogers 2003). Previous studies suggest that individual differences in preferences regarding the implementation process are associated with variation in the intent to participate (Cunningham et al. 2012, 2014). Aggregating the responses of professionals with disparate views can lead to inaccurate preference estimates while masking differences in preferences that might have allowed more targeted approaches to implementation (Powell et al. 2015).

## The Current Study

This study was conducted in the context of a program of research exploring the implementation of EBPs by mental health practitioners and educational professionals (Barwick et al. 2017; Cunningham et al. 2014). This research is based on the assumption that the preferences of those delivering mental health services influence the extent to which these programs are adopted, implemented, and sustained. As in previous studies of the implementation process (Cunningham et al. 2012, 2014), this study used a discrete choice conjoint experiment (DCE) to extend research on the preferences of service providers regarding the implementation of EBPs. These methods, used widely by marketing researchers (Orme 2009) and health economists (de Bekker-Grob et al. 2012), have been advocated as a method for selecting and tailoring implementation strategies that match the needs and preferences of service providers in different clinical contexts (Farley et al. 2013; Powell et al. 2015).

**Fig. 1:**
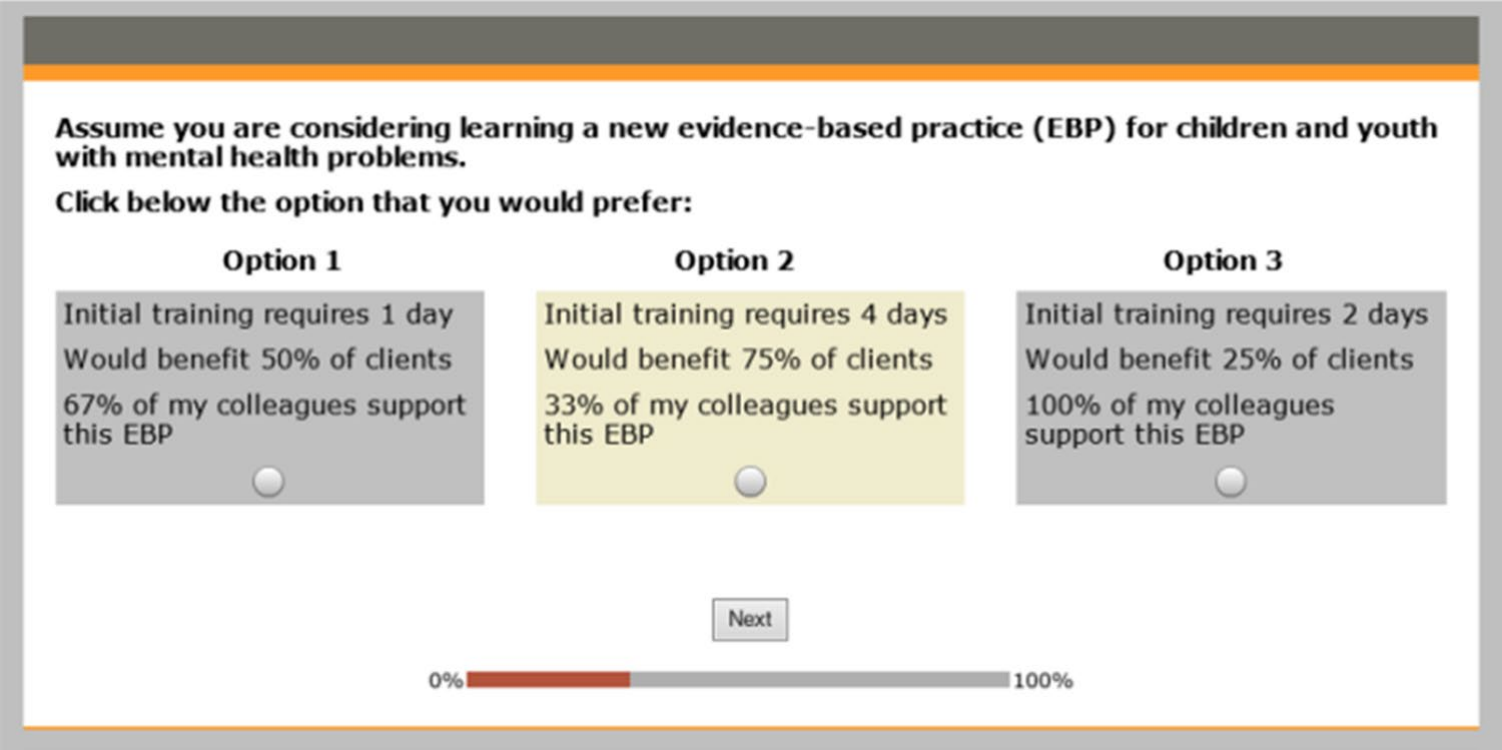
An example of the 18 choice tasks participants completed. Sawtooth Software’s experimental design module randomly assigned one of 999 versions of the survey to each participant

We began with a qualitative stage (focus groups) designed to identify dimensions of the implementation process that were relevant to service providers (Barwick et al. 2017). The themes from this stage were used to identify 14 EBP attributes of the implementation process. We included attributes focusing on the process of selecting EBPs, the social context in which implementation is conducted (e.g., supervisory or colleague support for an EBP), the organizational fit of EBPs, the training and implementation process (e.g. initial training options, training and follow-up time demands), and the outcome of EBPs (e.g., % of clients benefiting). Each attribute was described by four levels. Initial training, for example, could require 1, 2, 3, or 4 days. Using a design algorithm, we experimentally combined the levels of the study’s 14 implementation design attributes into sets of three hypothetical implementation options. Over a series of choice tasks, participants were asked to select the option they would prefer (Fig. 1).

DCEs can address several gaps in the research in this area. First, DCEs enable program developers and implementation teams to engage the stakeholders responsible for delivering or supervising EBPs at different stages of the implementation process (Aarons et al. 2011). Pre-implementation input allows planners to align training with organizational goals and issues, identify therapist preferences that might contribute to adherence failures that reduce effectiveness (Schoenwald et al. 2008), and decrease the number of individuals or agencies that discontinue implementation.

Second, rather than simply evaluating the components of a complex implementation process individually, DCEs approximate the complexity of the implementation decisions professionals actually make. Because each attribute level in a DCE is considered in the context of other attributes, the types of choice tasks presented in Fig. 1 encourage participants to consider the design tradeoffs decision makers will confront (de Bekker-Grob et al. 2012). Complex multi-attribute choices are more likely than simple ratings to elicit the heuristics influencing real world implementation decisions (Hauser 2014). Moreover, because participants make choices between *experimentally* manipulated combinations of implementation attributes, DCEs can estimate the relative influence of the individual components of complex implementation strategies on the intent to implement EBPs. Interestingly, although social desirability biases may influence the evaluation of organizational factors that predict attitudes toward EBPs (Izmirian and Nakamura 2016), complex choices in DCEs reveal latent preferences which may not be captured in simple rating scales or interviews (Caruso et al. 2009; Phillips et al. 2002).

Finally, when combined with latent class methods, DCEs can identify segments of participants who prefer different approaches to the implementation process (Hauber et al. 2016). Understanding differing views regarding the relative importance of different attributes of the implementation process is an issue of importance in service delivery systems where the front line staff who deliver EBPs, supervisors who support implementation, managers responsible for budgeting, and agencies that fund services bring different perspectives to the design process.

The EPIS model proposed by Aarons and colleagues points to stages in the implementation process at which a DCE might be applied (Aarons et al. 2011). During the Exploration Stage, for example, a DCE might be useful in estimating relative preference for different clinical problems (e.g., preschoolers with oppositional problems versus adolescents with anxiety disorders) or the therapeutic strategies an organization might pursue (e.g., a standard EBP or a more flexible approach based on elements). In a DCE focusing on the introduction of evidence-based strategies for improving mental health at school, most educators preferred training in school-wide strategies useful with all students rather than those targeting students with behavioral or emotional problems (Cunningham et al. 2014). During the adoption decision/preparation stage, a DCE could be used to engage stakeholders in a process examining different approaches to the selection of EBPs. Educators considering the implementation of school-based strategies to improve student mental health, for example, preferred programs chosen locally rather than by the Provincial Ministry of Education (Cunningham et al. 2014). The support of administrators, unions, and colleagues, coupled with both research and other schools finding a program works exerted a strong influence on the practice change strategy educators preferred (Cunningham et al. 2014). During the *Active Implementation* phase, a DCE might be useful in identifying opportunities to improve the fit between EBPs, organizational structure, clinical practices, client preferences, and implementation strategies. Educators, for example, preferred school-based mental health strategies that were compatible with their practice and closely linked to the provincial curriculum (Cunningham et al. 2014). During the *Sustainment* phase, a DCE might be useful in modeling a follow-up infrastructure that was consistent with user preferences. For example, although most educators preferred limiting follow-up training to two one-hour sessions, compensatory models suggest that this could be extended by ensuring that follow-up training included components educators valued: engaging experts, administrative support, a focus on skill acquisition and coaching, plus continuing efforts to enhance compatibility with existing practices (Cunningham et al. 2014).

The DCE used here addressed four research questions (RQ).

**RQ1. What attributes influence the decision to adopt EBPs?** We used a DCE to estimate the relative influence of 14 attributes of the implementation process on preference for hypothetical approaches to the implementation of EPBs.

**RQ2. Are there segments of children’s mental health professionals who prefer different approaches to the implementation of EBPs?** We used latent class analysis to identify segments of children’s mental health professionals with different preferences regarding the implementation process.

**RQ3. What demographic and professional characteristics are associated with membership in each segment?** We determined whether demographics, the intent to participate in implementation activities, and experience with EBPs were associated with segment membership.

**RQ4. What factors increase the intent to participate in the intensive training needed to ensure successful implementation?** In the absence of a comprehensive approach that includes active learning (observation, practice, and feedback), contextual support, and follow-up supervision, implementation programs often fail to yield therapeutic proficiency or to improve client outcomes (Beidas and Kendall 2010). We used randomized first choice simulations (Orme 2009) to model factors linked to the intent to engage in the more demanding training and follow-up that increases the likelihood that participants acquire the skill to adhere to protocols and deliver programs competently (Schoenwald et al. 2011).

## Method

### Participants

The study protocol was reviewed and approved by the Hamilton Integrated Research Ethics Board, the Hospital for Sick Children Research Ethics Committee, and the review committees of participating agencies. We approached 33 agencies providing publicly-funded community-based children’s mental health services in Ontario, Canada; 31 agreed to allow the study team to provide the survey link to their service providers. Among the 631 potential participants opening the link, 618 consented and 563 completed the entire survey. Informed consent was obtained from all individual participants included in the study. The anonymous survey collected no identifying information and did not record IP addresses.

## Attribute Development and Survey Design

The methods employed here have been described in a related study of the mental health implementation preferences of educators (Cunningham et al. 2014). Consistent with standard practice (Bridges et al. 2011) we derived attributes via a qualitative process designed to ensure that the survey reflected themes that were relevant to front line children’s mental health practitioners and the administrators who are critical to the implementation process (Barwick et al. 2017). Focus groups were conducted with 29 practitioners and 27 program supervisors from two large metropolitan areas in Ontario, Canada. Focus groups ranged in duration from 90 to 120 min. Using a semi-structured interview guide, interviewers explored factors influencing the decision to implement EBPs. Transcripts of these discussions, which were coded thematically, are the focus of a separate manuscript (Barac et al. 2017). Using a consensual approach, focus group themes were reduced to 14 attributes of the implementation process that were, in principle, amenable to change. To avoid a bias favoring attributes with a greater number of levels, each attribute included four levels ranging from low (e.g. 0% of my colleagues support this EBP) to high (100% of my colleagues support this EBP) (Wittink et al. 1990). A complete listing of attributes and attribute levels appears in Table 1.

**Table 1:** Utility coefficients and Z values for segments 1 and 2

Attribute	Latent class segment				Wald
	Segment 1		Segment 2		
Attribute levels	U	Z	U	Z	
Social context					
Supervisor support for EBP					8.24^a^
My supervisor does not support this EBP	−0.97	−4.11	−1.42	−16.54	
My supervisor supports this EBP 33%	−0.02	−0.12	−0.19	−3.29	
My supervisor supports this EBP 67%	0.12	0.78	0.59	10.47	
My supervisor supports this EBP 100%	**0.87**	**5.64**	**1.02**	**18.17**	
Colleague support for EBP					14.52^b^
0% of my colleague support this EBP	−0.81	−4.08	−1.59	−19.28	
33% of my colleagues support this EBP	−0.37	−2.18	0.09	1.56	
67% of my colleagues support this EBP	0.53	3.82	0.70	12.94	
100% of my colleagues support this EBP	**0.65**	**4.37**	**0.80**	**14.82**	
Trainers expertise and engagingness					32.13^c^
Trainer is not engaging nor an expert	−0.57	−3.04	−1.71	−18.37	
Trainer is engaging but not an expert	−0.09	−0.55	0.02	0.27	
Trainer is an expert but not engaging	−0.06	−0.39	0.01	0.11	
Trainer is an engaging expert	**0.72**	**3.50**	**1.68**	**26.60**	
Evidence of effectiveness					
Percent of clients benefiting					7.17
Would benefit 25% of clients	−0.92	−4.26	−1.49	−18.04	
Would benefit 50% of clients	−0.49	−2.64	−0.24	−4.03	
Would benefit 75% of clients	0.57	4.05	0.57	10.57	
Would benefit 100% of clients	**0.84**	**4.40**	**1.17**	**19.52**	
Effectiveness in other agencies					23.59^c^
This EBP is proven in research settings, but untested in agencies	−0.58	−3.01	−1.22	−15.60	
This EBP is proven in research settings and 1 agency	0.02	0.15	−0.32	−5.22	
This EBP is proven in research settings and 5 agencies	0.16	0.99	0.57	10.36	
This EBP is proven in research settings and 10 agencies	**0.40**	**2.93**	**0.96**	**17.31**	
Organizational fit of EBP					
Modifiability of EBP					57.37^c^
Modifications in this EBP are not allowed	−0.01	−0.06	−1.28	−13.71	
Minor modifications in this EBP are allowed	**0.26**	**1.80**	0.39	6.90	
Moderate modifications in this EPB are allowed	−0.11	−0.67	**0.75**	**12.92**	
Major modifications in this EBP are allowed	−0.14	−0.81	0.14	2.39	
Control over selection of EBPs					25.62^c^
Individual professionals select the EBP they will learn	−0.19	−0.94	0.18	2.90	
Individual programs within agencies select the EBP they will learn	**0.18**	**1.23**	**0.44**	**8.44**	
Individual agencies select the EBP they will learn	0.08	0.44	0.32	5.97	
Provincial ministry mandates the EBP professionals will learn	−0.07	−0.48	−0.94	−12.90	
Percent change to current practice					69.71^c^
Requires 25% change in current practice	−0.11	−0.69	**0.76**	**12.55**	
Requires 50% change in current practice	**0.07**	**0.52**	0.58	10.49	
Requires 75% change in current practice	0.02	0.10	−0.11	−1.91	
Requires 100% change in current practice	0.02	0.15	−1.23	−14.82	
Implementation process					
Training focus on knowledge versus skill					5.38
Training focuses 100% on knowledge	−0.82	−4.37	−1.15	−16.19	
Training focuses 67% on knowledge, 33% on step-by-step skills	0.02	0.11	0.39	7.17	
Training focuses 33% on knowledge, 67% on step-by-step skills	**0.68**	**4.24**	**0.66**	**12.01**	
Training focuses 100% on step-by-step skills	0.12	0.85	0.11	1.95	
Initial training via the internet					56.29^c^
No internet learning option	−0.80	−3.81	0.19	3.10	
33% of initial training can be completed online	−0.13	−0.75	**0.50**	**9.55**	
66% of initial training can be completed online	0.38	2.63	0.03	0.52	
100% of initial training can be completed online	**0.55**	**3.14**	−0.72	−10.44	
Active versus passive training process					19.18^c^
Participants don’t observe, practice, nor get feedback on new skills	−0.68	−3.37	−1.70	−18.10	
Participants observe new skills	−0.17	−1.09	−0.15	−2.48	
Participants observe and practice new skills	0.26	1.55	0.75	13.41	
Participants observe, practice, and get feedback on new skills	**0.60**	**3.65**	**1.11**	**18.86**	
Follow-up training					3.42
Includes 0 training follow-ups	−0.69	−3.26	−0.82	−12.12	
Includes a 1-day training follow-up	**0.38**	**2.65**	0.21	3.96	
Includes two 1-day training follow-ups	0.12	0.77	**0.41**	**7.38**	
Includes three 1-day training follow-ups	0.19	1.26	0.20	3.66	
Training group size					32.40^c^
I learn this alone	−0.06	−0.37	−0.84	−12.84	
I learn this in a group of 10	**0.24**	**1.60**	**0.89**	**16.32**	
I learn this in a group of 50	0.05	0.34	0.34	6.23	
I learn this in a group of 100	−0.23	−1.53	−0.39	−6.50	
Initial training time demands					4.50
Initial training requires 1 day	−0.23	−1.44	−0.03	−0.48	
Initial training requires 2 days	−0.05	−0.31	**0.19**	**3.58**	
Initial training requires 3 days	0.13	0.89	−0.01	−0.26	
Initial training requires 4 days	**0.15**	**1.01**	−0.15	−2.66	

Attributes are grouped consensually into those reflecting the social context, evidence of effectiveness, organizational Fit of EBPs, and implementation process. Attributes are ranked within each category in order of their importance to Segment 1. *U* parameter estimates expressed as zero-centered utility coefficients. Higher utility coefficients reflect a stronger preference. *Z* Z scores (U/SE). SE = U/Z. Within segments, the highest utility coefficient and Z value is bolded. Z values of 1.96 differ from zero (*p* < 0.05). ^a^ *p* < 0.05; ^b^ *p* < 0.01; ^c^ *p* < 0.001

## Other Measures

### Experience with EBPs

Using a 5-point Likert scale (1 = none, 5 = training and considerable experience), participants indicated their training and experience with 18 EBPs used in the field of children’s mental health (alpha = 0.82).

### Intent to Participate in Components of the Implementation Process

We created a brief 5-item Likert scale (alpha = 0.69) measuring willingness to participate (1 = strongly disagree, 3 = neither agree nor disagree, 5 = strongly agree) in different strategies to support the implementation of an EBP (e.g. let an expert observe my practice and give me feedback and tips).

## Procedure

After endorsing an electronic consent, reviewing a definition of EBPs, and practicing an introductory choice task, participants completed 18 experimental choice tasks systematically manipulating the levels of the study’s 14 attributes, as well as two hold-out choice tasks described below. Using Sawtooth Software’s experimental design module, we created a unique combination of attribute levels for each participant (Johnson et al. 2013). Each choice task presented three approaches to the implementation of EBPs (Fig. 1). The three options in each choice task were defined by the levels of three attributes. This partial profile design decreases the likelihood that participants would simplify choices (e.g. basing decisions on a single attribute level), rather than weighing the incremental value of all attributes in a profile (Patterson and Chrzan 2003). Participants were asked to: “Assume you are considering learning a new evidence-based practice (EBP) for children and youth with mental health problems. Click below the option you would prefer.” According to a main effects only design, no overlap in attribute levels was allowed (Orme 2009). The median time to complete the survey was 18.5 min.

## Data Analysis

Our approach to data analysis has been discussed elsewhere (Cunningham et al. 2012, 2014). Briefly, we used Latent Gold Choice 4.5 (Vermunt and Magidson 2005) to fit a latent class model to effects-coded choice data (Hauber et al. 2016). Using a maximum likelihood solution, latent class creates clusters (classes)—increasing homogeneity within classes while maximizing the distance between classes. We computed one, two, three, four, and five class solutions (Berlin et al. 2014; Lanza and Rhoades 2013). To reduce the likelihood of an unrepresentative model, each solution was computed ten times from a different point in the data (Berlin et al. 2014; Lanza and Rhoades 2013). The posterior probability of group membership was used to assign each participant to a specific class. A conditional logit model identified a set of parameter estimates fitting the choice data for each latent class (Vermunt and Magidson 2005). We calculated zero-centered utility coefficients reflecting relative preference for the levels of each attribute and importance scores quantifying the relative influence that variation in the levels of each of the study’s 14 attributes exerted on choices (Vermunt and Magidson 2005).

We used Sawtooth Software’s Randomized First Choice simulator to address RQ4 (Orme 2009). Using Latent Gold’s individual utility coefficients (Vermunt and Magidson 2005), the simulator predicted the percentage of participants likely to prefer different approaches to encouraging the implementation of EBPs (described in the "Results" section). The simulator’s algorithm assumes that, adjusting for two sources of error across 200,000 iterations, each participant would chose an option whose attribute levels yielded the highest combined utility (Orme 2009).

## Results

### Internal Validity

As described elsewhere (Cunningham et al. 2012, 2014), we included hold-out choice tasks at positions 6 and 14 in the sequence of choice tasks completed by each participant (Orme 2009). Although hold-out choice tasks were similar to the sample presented in Fig. 1, each participant viewed the same hold-out options. Responses to the two hold-out tasks were removed (e.g., held out) from the data prior to the computation of the utility coefficients in Table 1. We used Latent Gold’s individual utility coefficients (e.g., predicted preference for each attribute level), and Sawtooth Software’s Randomized First Choice Simulator (Orme 2009), to predict the percentage of participants likely to choose each of the three options in the two hold-out choice tasks. Next, using actual hold-out choices, we computed the percentage of participants choosing each of the three options in each hold out task (Vermunt and Magidson 2005). Mean absolute errors (MAE) were computed by averaging the absolute difference between the percentage predicted to choose, and the percentage actually choosing, the three options in each of the two hold-out choice tasks. With lower values reflecting better internal validity, MAEs of 3.4% for hold-out task one and 2.0% for hold-out task two suggest good internal or predictive validity (Orme 2009).

**RQ1. What attributes influence each segment’s decision to adopt EBPs?**

**RQ2. Are there segments of children’s mental health professionals who prefer different approaches to the implementation of EBPs?** Selecting a latent class solution requires the consideration of statistical fit, interpretability, and administrative utility (Berlin et al. 2014; Dziak et al. 2012; Hauber et al. 2016; Lanza and Rhoades 2013). Considering information criteria, sample size, and interpretability, we selected a two-class model (Table 2). A -2 Bootstrap log likelihood difference test confirmed that, in comparison to a one-class model, a two-class solution yielded a significant improvement in fit, -2LL Diff = 269.35, *p* < 0.001 (Vermunt and Magidson 2005).

**Table 2:** Fit indices for 1–5 latent class solutions

Measure	Number of latent classes				
	1	2	3	4	5
Parameters estimated	42	85	128	171	214
Degrees of freedom	521	478	435	392	349
Log-likelihood (LL)	−7741.23	−7606.56	−7472.18	−7368.01	−7285.20
Log-prior	−1.60	−2.60	−3.00	−3.27	−3.64
Log-posterior	−7742.83	−7609.16	−7475.19	−7371.29	−7288.85
AIC (based on LL)	15566.46	15383.12	15200.37	15078.03	14998.41
AIC3 (based on LL)	15608.46	15468.12	15328.37	15249.03	15212.41
BIC (based on LL)	15748.46	15751.45	15755.03	15819.02	15925.73
CAIC (based on LL)	15790.46	15836.45	15883.03	15990.02	16139.73
Entropy R^2^	1	0.692	0.691	0.693	0.736

*BIC* Bayesian information criterion, *AIC* akaike information criterion, *CAIC* consistent akaike information criterion. Entropy values range from 0 to 1 with higher values reflecting greater separation of classes. Vermunt considers an entropy value of 0.65 to be typical of the solutions reported in exploratory analyses (Vermunt 2010)

### Segment 1 (12%)

This segment’s choices indicated that they intended to pursue 100% of initial training online, devote more time to initial training (4 days), make only minor modifications to EBPs (Table 1), and participate in more implementation activities (see intent below). Importance scores (Table 3) showed the choices of this segment were highly sensitive to variation in the percentage of clients expected to benefit from the introduction of an EBP. The extent to which supervisors supported an EBP exerted a greater influence on this segment’s choices than any other attribute of the implementation process. The extent to which training focused on skill acquisition versus knowledge also exerted an important influence on their choices. In contrast, the amount of change in their practice an EBP might require, and the number of initial training days scheduled exerted relatively little influence on choices. Control over the selection of EPBs was less important than any other attribute (Table 3).

**Table 3:** Standardized importance scores for segments 1 and 2

Attributes	Latent class segment			
	Segment 1		Segment 2	
	R	I	R	I
Social context				
Supervisor Support for EBP	1	**12.9**	4	8.8
Colleague Support for EBP	4	**10.2**	5	8.7
Trainer’s Expertise and Engagingness	6	9.1	1	**12.3**
Evidence of effectiveness				
Percentage of Clients Benefiting	2	**12.3**	3	9.6
Effectiveness in Other Agencies	9	6.9	**6**	**7.9**
Organizational fit of EBP				
Modifiability of EBP	11	2.8	7	**7.4**
Control over Selection of EBPs	12	2.6	11	**5.0**
Percent Change to Existing Practice	14	1.3	8	**7.2**
Implementation process				
Training focus on skill versus knowledge	3	**10.4**	9	6.6
Initial training via the internet	5	**9.4**	13	4.4
Active versus passive training process	7	9.0	2	**10.2**
Follow-up training	8	**7.4**	12	4.4
Training group size	10	3.2	10	**6.3**
Initial training time demand	12	**2.6**	14	1.2

Attributes are grouped on a consensual basis into those reflecting social context, evidence of effectiveness, organizational fit of EBPs, and implementation process. Within each category, attributes are ranked in order of their importance to Segment 1. *R* Rank of each attribute’s importance within each segment, *I* relative importance of each attribute. Scores are expressed as percentages with the segment having the highest importance score bolded. Variations in the levels of attributes with higher importance scores exert a greater influence on implementation choices

### Segment 2 (88%)

This segment was intent on participating in fewer implementation activities (see below), preferred to devote half as much time to initial training (2 days), and make fewer changes to current practice (Table 1). This segment’s choices were moderately sensitive to the change in their current practice an EBP required (Table 3); they preferred EBPs minimizing change (Table 1). In comparison to Segment 1, this segment was less sensitive to the extent to which supervisors and colleagues supported an EBP. In contrast to Segment 1, trainer expertise and engagingness exerted a greater influence on this segment’s choices than any other attribute (Table 3). They were more sensitive than Segment 1 to the modifiability of EBPs (Table 3), preferring programs allowing moderate modifications (Table 1). Although they chose to limit initial training to 2 days, they preferred 2 days of follow-up training, twice as much as Segment 1.

### Converging Preferences

For both, preference for an EBP increased as a function of the number of agencies in which an EBP has been proven (Table 1). Both segments preferred EBP training conducted by engaging experts in groups of ten. Both preferred an active training process with observation, practice, and feedback on new skills. Both segments preferred that the decision to adopt an EBP be made by individual programs within agencies; they were least motivated to adopt a government-mandated EBP.

**RQ3. What demographic and professional characteristics are associated with membership in each segment?** Segment 1, *M* = 4.04 *SD* = 0.63, reported a greater intent to participate in implementation activities, *F* (1, 561) = 8.004, *p* = 0.005, than did Segment 2, *M* = 3.82 *SD* = 0.61. The total EBP experience scores reported by Segment 1 (*M* = 31.5, *SD* = 13.6) and Segment 2 (*M* = 29.6, *SD* = 9.0) did not differ, *t* (561) = −1.50, *p* = 0.13. On average, Segment 1 reported training and experience with 4.2 (*SD* = 3.1) of a list of 18 EBPs. Segment 2 reported training and experience with 3.8 EBPs (*SD* = 4.3). Table 4 shows that age, educational level, practice setting, and years of experience were not associated with segment membership. Although there is significant variation associated with professional backgrounds, small samples in some groupings make this difficult to interpret.

**Table 4:** Demographic percentages for participants in segments 1 and 2

	Latent class segment				
	N	%	% Segment 1	% Segment 2	χ2
Sample size	563	100	12	88	
Age					3.72
18–29	88	15.6	9.1	90.9	
30–39	188	33.4	13.3	86.7	
40–49	127	22.6	15.7	84.3	
50–59	119	21.1	9.2	90.8	
60 and older	41	7.3	9.8	90.2	
Gender					3.83
Female	455	80.8	10.8	89.2	
Male	108	19.2	17.6	82.4	
Education					7.65
Graduated from college or less	170	30.2	17.6	82.4	
Bachelor’s degree (BA or BSc)	132	23.4	11.4	88.6	
Master’s degree	238	42.3	8.8	91.2	
Doctoral or medical degree	23	4.1	8.7	91.3	
Education background					25.97*
Social work	224	39.8	7.1	92.9	
Child and youth worker and ECE	169	30.0	16.6	83.4	
Psychology/psychiatry/other medical training	102	18.1	11.8	88.2	
Education	11	2.0	27.3	72.7	
Nursing	7	1.2	42.9	57.1	
Administration	3	0.5	66.7	33.3	
Other	47	8.3	8.5	91.5	
Practice setting					1.20
Outpatient children’s mental health service	361	64.1	11.1	88.9	
Inpatient, residential, day treatment	126	22.4	14.3	85.7	
Educational	65	11.5	13.8	86.2	
Hospital	11	2.0	9.1	90.9	
Experience					1.59
5 years or less	132	23.4	12.9	87.1	
6–15 years	204	36.2	13.7	86.3	
16–25 years	138	24.5	10.9	89.1	
26 years or more	89	15.8	9.0	91.0	

*p < 0.001

**RQ 4. What factors increase the intent to participate in the intensive training and change in practice needed to ensure successful implementation?** Using randomized first choice simulations (Orme 2009), we estimated the percentage of participants in each segment likely to prefer the more demanding training, follow-up, and change in practice needed to ensure the successful implementation of EBPs (Beidas and Kendall 2010; Herschell et al. 2010). We simulated two approaches to implementation. According to the basic training model, participants received (1) 2 days of initial training and (2) 2 days of follow-up training, focusing (3) on 33% skills and 67% knowledge, and requiring (4) a 25% change in practice. The enhanced training model required (1) 4 days of initial training, (2) 4 days of follow-up training, focusing (3) 67% on skills 33% on knowledge, and a (4) 50% change in practice. Table 5 shows that overall, 76.6% of participants preferred the less demanding Basic Training Model. Although 50.8% of Segment 1 was predicted to prefer the Enhanced Training Model, only 19.7% of Segment 2 would choose this option.

**Table 5:** Randomized first choice simulations: percentage of participants in each segment predicted to prefer different approaches to the implementation of evidence-based children’s mental health practices

Sensitivity analysis on support by administrators	Latent class segment					
	Total sample		Segment 1		Segment 2	
Approach to implementation	%	(SE)	%	(SE)	%	(SE)
Simulation 1						
Basic implementation	76.6	(0.5)	49.2	(0.8)	80.3	(0.1)
Enhanced implementation	23.4	(0.5)	50.8	(0.8)	19.7	(0.1)
Simulation 2						
Basic implementation	51.9	(0.4)	27.8	(0.6)	55.3	(0.2)
Enhanced implementation +33% supervisor support	48.1	(0.4)	72.2	(0.6)	44.7	(0.2)
Simulation 3						
Basic implementation	35.7	(0.2)	23.6	(0.3)	37.4	(0.1)
Enhanced implementation +67% supervisor support	64.3	(0.2)	76.4	(0.3)	62.7	(0.1)
Simulation 4						
Basic implementation	26.1	(0.2)	12.9	(0.3)	27.9	(0.1)
Enhanced implementation +100% supervisor support	73.9	(0.2)	87.1	(0.3)	72.1	(0.1)

Utility coefficients and importance scores suggest that a shift in preference for the enhanced training model could be accomplished by increasing supervisor support. Simulation 2 varied the level of supervisor support for Enhanced Training from a non-supportive supervisor to 100% supervisor support. Table 5 shows that, as supervisor support increased to 100%, predicted preference for the enhanced training model increased from 50.8 to 87.1% of Segment 1 and 19.7 to 72.1% of Segment 2.

## Discussion

This study makes three contributions to the study of the implementation of evidence-based children’s mental health services. Methodologically, we illustrate the application of a set of preference modeling methods which, while used widely by marketing researchers (Orme 2009) and health economists (de Bekker-Grob et al. 2012), have rarely been extended to inform the implementation of evidence-based mental health practices. Second, the implementation of EBPs is a complex process (Beidas and Kendall 2010; Damschroder et al. 2009). This study illustrates the importance of understanding the relative impact of the many factors influencing the adoption and implementation process (Beidas and Kendall 2010). Understanding these factors could contribute to the design of more successful implementation plans (Powell et al. 2015). Third, latent class analysis shows the importance of understanding individual differences in implementation preferences and illustrates the potential of these methods as an approach to tailored implementation planning (Powell et al. 2015). Below, we consider the implementation preferences of participants in the two segments, discuss design preferences these segments have in common, and consider the broader implications of our findings.

### Segment 1 (12%)

Given administrative support and an EBP expected to benefit a significant proportion of their clients, this segment was intent on investing more time in the initial training process. They evidenced the openness to change that has been linked to the use of EBPs (Beidas et al. 2015); they were willing to make greater change to their own practice and intent on making fewer changes to EBPs. This segment is similar to, though much smaller than, the change ready segment in a previous study of the mental health practice change preferences of educators (Cunningham et al. 2014).

### Segment 2 (88%)

Segment 2 was less intent on participating in the implementation process. In comparison to Segment 1, they preferred completing a lower percentage of initial training online and devoting fewer days to the initial training process. Decision control was more important to this segment. They preferred making fewer changes in their own practice but more significant modifications to EBPs. Although this segment is much larger than the demand sensitive educators from a previous study (Cunningham et al. 2014), their implementation preferences are very similar.

## Implications

### Manage the Social Context

The influence of the social context on implementation preferences was striking. Participants preferred EBP training delivered by engaging experts, supported by supervisors, and backed by their colleagues. These findings are consistent with previous studies (Herschell et al. 2014; Palinkas et al. 2008; Rosen et al. 2016). Colleagues and supervisors exert a similar influence on the implementation preferences of educators (Cunningham et al. 2014) and addiction professionals (Cunningham et al. 2012). These findings are consistent with evidence regarding the importance of leadership engagement (Damschroder et al. 2009), the influence of opinion leaders (Doumit et al. 2007; Flodgren et al. 2011; Schoenwald et al. 2013), and with broader studies of the effect of organizational climate on the implementation of EBPs (Aarons et al. 2012). Indeed randomized trials suggest that the inclusion of a focus on strategically important organizational processes can improve the outcome of EBPs (Glisson et al. 2010).

Ambivalence regarding the adoption of EBPs may be stronger in clinical settings in which practitioners hold a range of theoretical perspectives (Beidas and Kendall 2010). Our findings, however, suggest that the successful introduction of an EBP requires a process ensuring that both supervisors and colleagues consistently support implementation. Colleagues who are members of Segment 1, for example, might serve as “champions” or key opinion leaders (Schoenwald et al. 2013) who support implementation by advocating on behalf of an EBP, generating enthusiasm, and diffusing the push-back organizational change may elicit (Damschroder et al. 2009).

### Enhance Supervisory Processes

Supervisor support exerted an important influence on the EBP choices of both segments. Although simulations predicted that few participants (23%) would prefer an enhanced approach to implementation that required more training, follow-up, and changes to current practice, with increasing supervisor support, most participants (73.9%) would prefer a level of intensity approaching that needed to implement EBPs with the integrity required to improve client outcomes (Beidas and Kendall 2010). Several studies and a number of the attributes included here, point to components of supervision that might contribute to the relative importance of this dimension of the implementation process. In the Child STEPS project, for example, ethnographic methods suggested that the quality of the relationship between supervisors and therapists was associated with short-term implementation of EBPs (Palinkas et al. 2008). A study of 57 therapists and 12 supervisors concluded that, when supervisors modeled components of EBPs and therapists rehearsed these skills in role-play scenarios, the type of active learning strategies that were important to our study’s participants, therapists were more likely to use these strategies in subsequent sessions (Bearman et al. 2013). In a 1-year follow-up of 1979 youth and their families receiving multi systemic therapy from 429 therapists, clinicians whose supervisors emphasized adherence to the principles of MST reported greater adherence (Schoenwald et al. 2009). When supervisors adhered to the study’s supervisory protocols, and encouraged the development of clinicians, caregivers reported greater reductions in youth externalizing problems (Schoenwald et al. 2009). Schoenwald and colleagues summarized much of the extant research addressing the role of supervision in the implementation of EBPs (Schoenwald et al. 2013). Using MST protocols as a model, they developed a supervisory approach to support the implementation of Links to Learning, a comprehensive mental health intervention for JK to grade 4 students with behavioral problems. The model included a focus on implementing the universal (peer assisted learning) and targeted (daily report card) components of the intervention, solving case and system related problems, developing specific action plans, building both therapist skills and supervisor skills, identifying adjunct services, and considering the organizational context in which Links to Learning was delivered (Schoenwald et al. 2013).

### Provide Supporting Evidence

Evidence of effectiveness is critical to professional decisions regarding the adoption of EBPs (Herschell et al. 2014; Rosen et al. 2016). Participants preferred EBPs proven in both research and real world agency applications. As in a previous study, implementation choices were particularly sensitive to the percentage of clients expected to benefit (Cunningham et al. 2012). Willingness to adopt EPBs also increased as a linear function of the number of agencies that had implemented the program successfully. These findings suggest that, in addition to efficacy studies evaluating the performance of an intervention under optimal circumstances, efforts to introduce EBPs should be supported by a significant body of evidence regarding their feasibility and effectiveness in real-world clinical applications (Damschroder et al. 2009; Revicki and Frank 1999).

### Enable Local Decision Control

Most participants preferred EBPs selected by programs within agencies rather than the governments who fund services. Engaging those responsible for the conduct of an EBP in the selection process may enhance a sense of ownership that supports long-term stability (Powell et al. 2015). As local decision control exerted more influence on the choices of Segment 2 than Segment 1, it may be particularly important to engage this segment in discussions regarding the process of implementing EBPs. The importance of decision control is consistent with previous implementation research (Cunningham et al. 2012, 2014) and a wider body of evidence supporting the importance of personal agency in the organizational change process (Cunningham et al. 2002). Randomized trials have linked therapist perceptions of decision control to improved clinical outcomes (Schoenwald et al. 2008).

### Create Flexible Approaches to Implementation

Latent class analyses identifying segments with differing preferences emphasize the importance of a flexible approach to the implementation of EBPs. This might include varying the opportunity to complete preliminary training online, the pace at which the components of EBPs are introduced (Bernstein et al. 2015; Chorpita and Daleiden 2009; Weisz et al. 2012), or the amount of follow-up training and support which is available. Importantly, the demands of an implementation process based on Segment 1’s preferences, a small group who might be expected to assume an influential role in the introduction of EBPs, might exceed the commitment of Segment 2, a majority of the study’s participants. Flexible approaches which can be adapted to existing work-flows are more likely to be adopted (Damschroder et al. 2009; Rosen et al. 2016), an important point given this segment’s size (88%).

### Optimize Training

Consistent with the recommendations of a large body of implementation research (Bearman et al. 2013; Beidas and Kendall 2010; Herschell et al. 2010, 2014), both segments preferred an active training process providing small group (N = 10) opportunities to observe, practice, and receive feedback on new skills. Simulations, however, predicted that strong supervisor support would be needed to engage Segment 2 in the intensive multi-component training likely to achieve the client outcomes they valued (Beidas and Kendall 2010; Herschell et al. 2010). Their sensitivity to the expertise and engagingness of trainers, and this segment’s size, emphasize the importance of high quality training teams.

### Develop Online Options

Both segments were willing to complete some initial training online. Indeed, Segment 1 preferred to complete all initial training online. Although online training may provide necessary but not sufficient background knowledge (Herschell et al. 2010), delivering components of the implementation process via an e-learning format could provide the flexibility to fit training into individual workflows, adjust training to segments with different learning needs and objectives, and reduce costs (Herschell et al. 2010).

### Manage Local Adaptations

Both segments were more likely to choose EBPs allowing local modification. This is consistent with evidence that service providers prefer an approach allowing the flexible application of the components of EBPs (Chorpita et al. 2015). The extent to which programs allowed modification was twice as important to Segment 2, 88% of the study’s participants. Engaging those responsible for the conduct of an EBP in the process of adaptation may allow the fit needed to increase professional commitment (Palinkas et al. 2008) and enhance a sense of ownership that encourages long-term stability (Stirman et al. 2012). Nonetheless, the tendency of therapists to overestimate their acquisition of EBP skills (Beidas and Kendall 2010), Segment 2’s preference for more limited initial training, and Segment 1’s preference for minimal follow-up training, emphasize the importance of balancing user preferences with evidence regarding the level of training needed to implement EBPs with the integrity required to enhance outcomes (Beidas and Kendall 2010; Powell et al. 2015; Schoenwald et al. 2008). Given limited knowledge regarding the impact of fidelity consistent and inconsistent modifications of EBPs (Stirman et al. 2013, 2015), this issue merits caution and further study.

## Limitations

The results of this study should be interpreted in the context of several limitations. First, this research was conducted in Canada. As publicly funded children’s mental health service providers, the organizations from which participants were selected face different funding processes than those in the United States (Hoagwood et al. 2014). The extent to which these findings might generalize to other settings is unclear.

Second, the average participant in this study reported training and experience with approximately four EBPs, a factor that was not linked to segment membership. It should be noted, however, that our sampling strategy approximates those studying the attitudes of community therapists toward EBPs (Beidas et al. 2015; Nelson and Steele 2007) rather than those providing all participants formal training and supervision in a single EBP or a fixed set of EBPs (Chorpita et al. 2015; Palinkas et al. 2013).

Third, although we recruited a large sample, can determine the percentage of agencies approached who agreed to participate (94%), and recorded the proportion of individuals opening the link who completed the survey (89%), we are unable to determine the larger number who did not receive, or failed to open, the survey.

This cross sectional study is similar to many marketing research and health economic applications administering DCEs early in the product and service design cycle. Although this study does not capture the shift in attitudes that may occur with training and exposure to EBPs (Palinkas et al. 2013), administering cross sectional DCEs early in the design process allows results to inform planning, enables implementation to be tailored to local contexts (Powell et al. 2015), and allows planners to explore design options that extend existing practices. Finally, although we included a relatively large number of attributes, our models are limited by attributes that were not included in the survey.

## Conclusion

Mental health practitioners are more likely to adopt EBPs that are supported by colleagues and supervisors, benefit a significant number of clients, and are backed by evidence of effectiveness in other agencies. They prefer that engaging experts conduct small group, skill-focused, active learning with follow-up training. They value program-based decisions and the opportunity to introduce local modifications. Securing the participation of an entire organization requires a flexible approach considering the preferences of segments committed to participating in the intensive approaches needed to ensure successful implementation as well as those who may prefer less change in their practice, fewer training days, and more opportunities to introduce local modifications.
